# Editorial: Emerging unconventional plants for derived food products and ingredients

**DOI:** 10.3389/fnut.2024.1373439

**Published:** 2024-02-23

**Authors:** Maria Inês Dias, Rúbia C. G. Corrêa, Nadia Cristiane Steinmacher, José Pinela, Carla Pereira

**Affiliations:** ^1^Centro de Investigação de Montanha (CIMO), Instituto Politécnico de Bragança, Campus de Santa Apolónia, Bragança, Portugal; ^2^Laboratório Associado para a Sustentabilidade e Tecnologia em Regiões de Montanha (SusTEC), Instituto Politécnico de Bragança, Campus de Santa Apolónia, Bragança, Portugal; ^3^Post-Graduate Program in Clean Technologies, Cesumar Institute for Science, Technology and Innovation—ICETI, Cesumar University—UNICESUMAR, Maringá, Brazil; ^4^Departamento Acadêmico de Alimentos (DAALM), Universidade Tecnológica Federal do Paraná, Medianeira, Brazil

**Keywords:** innovative food products, unconventional edible plants, wild food plants, human nutrition, bioactive compounds, alternative diets, food security

The “Emerging Unconventional Plants for Derived Food Products and Ingredients” *Frontiers in Nutrition* Research Topic aimed to address innovative solutions to ensure food security, such as nutrient-rich alternatives, sustainable agriculture, and advanced industrial technologies, by exploring Unconventional Edible Plants for developing new, nutritious, and safer food products. This Topic falls in the 2030 Sustainable Development Agenda, driven by societal, economic, and environmental factors, to eliminate world hunger and poverty. Research on sustainable agriculture, nutritional profiling, and innovative food development holds promise in integrating these unconventional plants into daily base diets worldwide, a perspective that has been shared in academia, not only for food purposes (1) but also medicine (2). The present Research Topic gathered 41 authors from China, Israel, India, Egypt, and Saudi Arabia that provided in-depth scientific knowledge regarding the potentiality of unconventional plants to be applied as functional ingredients in food and pharmaceutical formulations. The use of Unconventional Plants has been carried out for hundreds of years by local populations, who, knowing their attributes, use them in their daily lives for various purposes. By applying state of the art technologies and methodologies in the studies mentioned above, we have clear examples of the practicality of this topic at an economic level, but above all at a social level.

The first manuscript published in the Research Topic was the extensive review article by Fatima et al. that gathered researchers from India, Egypt, and Saudi Arabia, and focused on the description of *Bacopa monnieri*, an herb with a rich history in Ayurvedic medicine. This plant has been recognized for its diverse therapeutic properties, including its role as a memory enhancer, sedative, and anti-epileptic agent. The high added-value extracts isolated from *B. monnieri*, rich in flavonoids, saponins, and triterpenes, have demonstrated the ability to prevent oxidative, mitochondrial, and endoplasmic reticulum stress, thereby influencing the aging process in *C. elegans*. This review article specifically explored its potential in combating cancer and neurodegenerative diseases by examining several clinical studies on the phytochemistry and pharmacological applications of *B. monnieri*. The manuscript delved into the mechanisms underlying its action in various types of cancer and its therapeutic implications, the molecular mechanisms involved in therapeutic interventions, potential toxicities and safety concerns, and synergistic potentials in cognition and neuroprotection. The authors also highlighted the significance of specific *B. monnieri* constituents, including Bacoside A, Bacoside B, Bacosaponins, and Betulinic acid, in conferring neuroprotection. These bioactive components exhibit neuroprotective properties by reducing reactive oxygen species (ROS), mitigating neuroinflammation, inhibiting amyloid-β aggregation, and enhancing cognitive and learning behavior. Among the major phytoconstituents of *B. monnieri* are saponins such as bacoside A3, bacopaside II, X, and bacopasaponin C and its isomer. Noteworthy findings reveal the inhibitory effects of bacosides on the viability and proliferation of glioma cells, suggesting a promising anti-cancer activity, particularly in the treatment of glioblastoma (3). In conclusion, this review underscores the potential of *B. monnieri* extracts in the treatment of neurological disorders, including Alzheimer's disease. However, it is essential for future investigations to compare the neuroprotective effects of *B. monnieri* extracts with standard drugs, establishing a foundation for systematic clinical applications in the field of neuroscience.

The manuscript by Wang et al. involved researchers from China, and deal with the utilization of jasmine flower residue as an unconventional feed for goats. This residue was investigated, focusing on its impact on meat quality and flavor. The study involved 24 castrated Nubian male goats, randomly assigned to two groups: one fed a mixed diet containing 10% of jasmine flower residue and the other receiving a conventional diet for a period of 45 days. The addition of flower residue to the goat diet resulted in notable improvements in meat quality. Specifically, the Shear force of the *longissimus dorsi* muscle decreased, indicating increased tenderness. Additionally, the cross-sectional area and diameter of muscle fibers were reduced, further enhancing meat quality. The study revealed that flower residue supplementation increased the content of glutamic acid and ω-3 polyunsaturated fatty acids (PUFAs), such as C18:3n3 and C20:5N3, while decreasing the content of C24:1 and saturated fatty acids (C20:0 and C22:0). The flower residue also influenced the flavor profile of the meat, increasing the content of acetaldehyde and hexanal. The introduction of new volatile components in the meat contributed to improved umami, saltiness, and richness. The study also identified changes in the volatile composition of meat, with significant differences between the two group studies. Electronic nose and electronic tongue analyses supported these findings, indicating increased aromatic components and enhanced umami and richness in the jasmine flower residue-fed goat meat group. The study emphasized the potential economic efficiency and reduced feeding costs associated with the use of unconventional feeds in goat breeding. The findings suggest that jasmine flower residue supplementation not only improves meat quality and flavor but also enhances the nutritional value of goat meat by increasing amino acids and ω-3 PUFAs. In conclusion, this study provides groundbreaking evidence that dietary supplementation with jasmine flower residue can significantly enhance the tenderness, nutritional profile, and flavor of goat meat. The results open up new possibilities for efficient utilization pathways for jasmine flower, offering valuable insights for future studies aiming to improve meat quality and flavor through dietary interventions in animal breeding.

Zhou et al. focused on the potential anti-obesity effects of bamboo shoot dietary fiber (BSDF) and the underlying mechanisms, considering the crucial role of gut microbiota in obesity development. After a 12-week intervention with BSDF in high-fat diet (HFD)-fed mice, the researchers evaluated obesity-related phenotypic indicators, conducted liver transcriptomic analysis, examined changes in gut microbiota through 16S rRNA gene sequencing, explored BSDF's impact on gut microbiota metabolites, and validated the significance of gut microbiota using an antibiotic animal model. The results demonstrated that BSDF effectively reduced lipid accumulation in the liver and adipose tissue, alleviated dyslipidemia, and improved insulin resistance in HFD-fed mice. Liver transcriptome analysis revealed that BSDF modulated peroxisome proliferator-activated receptor (PPAR) and fatty acid metabolic pathways, improving lipid metabolism and liver injury. Analysis of gut microbiota composition showed that BSDF significantly enriched beneficial bacteria such as *Bifidobacterium, Akkermansia, Dubosiella*, and *Alloprevotella*. Fecal metabolomics and gut microbiota metabolites revealed increased levels of short-chain fatty acids (SCFAs) and enriched bile acids, contributing to improved lipid metabolism. Importantly, when gut microbiota was abrogated through antibiotic treatment, the beneficial effects of BSDF on obesity-related metabolic disorders were invalidated, highlighting the pivotal role of gut microbiota in BSDF's effects. The study proposes that BSDF, acting as a prebiotic supplement, has the potential to improve obesity by modulating the gut microbiota and influencing host PPAR and fatty acid metabolic pathways. The research provides comprehensive insights into the beneficial effects of BSDF, emphasizing its potential as a dietary resource for developing interventions against obesity and metabolic diseases. The investigation not only assessed the phenotypic improvements associated with BSDF supplementation in HFD-fed mice but also delved into the molecular mechanisms. The transcriptomic analysis identified the PPAR signaling pathway as a key metabolic pathway influenced by BSDF. Furthermore, changes in gut microbiota composition and metabolites demonstrated the intricate relationship between BSDF, gut microbiota, and host metabolism. In conclusion, this study contributes valuable knowledge regarding the potential of BSDF in addressing obesity and associated complications. The findings encourage further exploration of the prebiotic value of BSDF in developing dietary interventions for obesity and metabolic diseases.

Finally, the study of Li et al. delved into the structural and physicochemical properties of bracken fern (*Pteridium aquilinum*) starch, a lesser-explored starch in the scientific literature. Two bracken starch samples were systematically investigated, revealing key characteristics that can contribute to their potential utilization in both food and non-food industries. The amylose contents of the two bracken starches were found to be 22.6% and 24.7%, respectively. The starch granules exhibited near-spherical, ellipsoidal, or prolate ellipsoidal shapes, with a mean area diameter <20 μm. X-ray diffraction (XRD) analysis indicated a typical C-type crystalline structure for bracken starches. Notably, during the gelatinization event, these starches displayed lower viscosity compared to typical rice starch and lower gelatinization temperatures than those observed in cereal starches. Furthermore, the gelatinized bracken starches formed softer and stickier gels in comparison to rice and potato starches with similar amylose contents. The molecular weight and branching degree of bracken starches were significantly higher than starches from many other sources, showcasing unique structural characteristics. The branch chain length distribution revealed structural similarities between bracken starches and certain rice varieties. Importantly, the study highlighted distinct differences between the two bracken starch samples in terms of amylose content, gel hardness, gelatinization temperature, and other structural properties. While the research provides valuable insights into the utilization of bracken starch in various industries, it emphasizes the need for further investigation into the genetic diversity and the impact of growing environments on the properties of bracken starch. In conclusion, this study not only expands our understanding of bracken starch but also lays the groundwork for its potential applications in food and non-food industries. The unique physicochemical properties observed in bracken starches suggest opportunities for the development of novel products, warranting continued exploration and research in this field.

The four manuscripts featured in this Research Topic provide valuable insights, unlocking the vast potential of Unconventional Food Plants. These contributions serve as a promoter for expanding global research endeavors, focusing on the economic and societal advantages associated with harnessing unconventional plant resources across diverse applications. The knowledge derived from these publications is aimed to inspire and fuel further exploration into the multifaceted benefits offered by unconventional food sources on a global scale.

## Author contributions

MD: Writing – original draft, Writing – review & editing. RC: Writing – original draft. NS: Writing – original draft. JP: Writing – review & editing. CP: Writing – review & editing.
